# A novel assessment of self-perceptions of recovery from illness and injury in U.S. Navy service members

**DOI:** 10.3389/fresc.2026.1846239

**Published:** 2026-09-09

**Authors:** Lisa M. Hernández, Nikita R. Tovey, Anu Venkatesh, Kristin J. Heaton, Justin S. Campbell, Jessica M. Hru, Marcus K. Taylor

**Affiliations:** 1 Warfighter Performance Department, Naval Health Research Center, San Diego, CA, United States; 2 Leidos, Inc., San Diego, CA, United States; 3 Naval Information Warfare Center Pacific, Cyber Science & Technology, San Diego, CA, United States; 4 Military Performance Division, United States Army Research Institute of Environmental Medicine, Natick, MA, United States; 5 Marine Corps Forces Reserve, New Orleans, LA, United States; 6 Johns Hopkins University, Baltimore, MD, United States

**Keywords:** injury, psychometrics, questionnaires, rehabilitation, resilient responses, stress immunity

## Abstract

**Objective:**

There is no measure that assesses self-perceptions of recovery in service members. This report describes the development and validation of the Military Illness and Injury Recovery Scale-17 (MIIRS-17) in a sample of active duty personnel.

**Methods and measures:**

Participants (*N* = 182) were U.S. Navy aircrew who responded to a survey including 19 candidate scale items and measures of theoretically relevant correlates (e.g., pain). Factor structure of the scale was explored with principal components analysis. Convergent validity was established with bivariate associations between selected correlates, total recovery, and subscales. A multiple linear regression model was used to explore the contributions of each predictor (e.g., fatigue) for total recovery. Models were age-adjusted, as applicable.

**Results:**

The final 17-item scale was highly consistent with four subscales, which we termed immune recovery, musculoskeletal injury recovery, physical recovery, and rehabilitation response. Total recovery and relevant correlates were related in the expected directions. In the combined model, eight predictors and age explained 39.6% of variance in total recovery. Sleep disruption, resilience, and age, independently contributed to total recovery.

**Conclusion:**

Our preliminary results indicate that the MIIRS-17 is a valid and reliable instrument for assessing self-perceptions of recovery in military members.

## Introduction

In military environments, service members are challenged by multiple stressors including physical demands (1), environmental extremes (2), and psychological trauma (3). These exposures can become catalysts for illness (4) and/or can cause injury (5). Force readiness depends upon how rapidly military personnel can bounce back from stressors (e.g., injury, intense training, deployments). Recovery from illness or injury is influenced by internal and external factors such as physical fitness (6), psychological factors (7), and the availability/accessibility of rehabilitation resources (8). Additionally, self-perceptions about recovery play an important role in determining propensity to recover. This is understudied in service members, so it is crucial to investigate self-perceptions about recovery to improve adaptability and lethality.

The literature about self-perceptions of recovery is limited to a small number of studies in injured, civilian patients (9–12). From these studies, two novel, subjective (self-report) tools have been developed to support clinical practice. One scale measured injury perceptions in a mixed sample of students and physical therapy clinic patients (9). That study found several types of perceptions that should be used to provide individualized rehabilitation care and treatment. The second tool measured self-perceptions in rehabilitation patients who had sustained a traumatic brain injury [TBI (11)]. The authors asserted that this measure could indicate patient readiness to engage in occupational therapy sessions and flag potential impediments for progress. Evidence of preliminary validity and reliability was reported for both measures, demonstrating the potential for quantitative assessments of self-perceptions to be used in a clinical setting. Another group found that self-perceptions predicted time to return to work after rehabilitation and therefore, self-perceptions were indicators of risk for disability (10). Altogether, this body of work reinforces the importance and impact of self-perceptions on recovery.

Due to the nature of military operations and the higher probability of life-threatening events within these environments, research about service member recovery is extensive. However, the literature tends to focus on interventions and objective measurements of recovery. In these studies, subjective measures are often used in conjunction with recovery interventions. For example, self-report measures can indicate risk factors for delayed recovery and positive recovery outcomes following musculoskeletal injury [MSKI (13)]. In an exploratory study of United Kingdom Armed Forces personnel, investigators used a self-report measure to determine if a period of rest and recuperation supported recovery following deployment (14). In addition, subjective measures of psychological traits have supported the important role of self-efficacy in rehabilitation responses for post-concussive injuries (15) and posttraumatic recovery (16), and of resilience (a person's ability to overcome adversity) as a predictor of recovery outcomes (17). While subjective measures can support recovery interventions and the prediction of recovery outcomes, there are currently no measures that assess self-perceptions of recovery in service members.

In this study, we describe the development and initial validation of the Military Illness and Injury Recovery Scale-17 (MIIRS-17), a quantitative measure of recovery self-perceptions. We expected to produce an overall, internally stable scale with moderately correlated subscales that corresponded to, and were differentiated by, recovery from various types of insults (e.g., illness, MSKI, intense exercise) and self-perceptions of responses to rehabilitation. Initial convergent validity was established by exploring the associations between recovery and theoretically relevant correlates. We anticipated that 1) MIIRS-17 recovery scores would be inversely associated with pain, fatigue, occupational stress, mental health symptoms, and sleep disruption, 2) recovery scores would be positively related to resilience and military readiness, and 3) that age would emerge as a covariate in some, or all, of these relationships.

## Materials and methods

### Design

This was a cross-sectional study with no exclusion criteria, and participants who volunteered to enroll in the study provided their verbal consent. In a single, in-person session, participants completed a survey on a computer tablet using a non-identifiable study identification number (e.g., 001). The survey included questions about the participant's background (e.g., age, military experience), the MIIRS-17, and measures of theoretically relevant correlates (detailed in data collection section).

### Participants

The Naval Health Research Center Institutional Review Board reviewed and approved this study protocol (NHRC.2023.0005). We recruited participants (*N* = 182) from U.S. Navy aircrew squadrons under Helicopter Sea Combat Wing Pacific (San Diego, CA) and Fleet Logistics Multi-Mission Wing (San Diego, CA). All active duty personnel from the aforementioned commands were eligible and invited to a recruitment session during which a brief description of the study was provided. Those who were interested in participation were invited to stay for the informed consent process. Participants were given the options to skip questions that they did not want to answer and to discontinue their study involvement at any time; however, no one withdrew after study enrollment.

### MIIRS-17 development

Scale items were developed by a team of three health and human performance scientists, including one expert in scale development and psychometrics who is also a military veteran. For this process, the team used our operational definition of recovery: one's ability to regain stable conditions—typically through intervention (e.g., rest, rehabilitation)—after a significant disruption, alteration, or injury. Based on this definition, we aimed to address the lack of a validated instrument that measures self-perceptions of recovery in military personnel. Following a review of the limited, relevant literature, and in conjunction with our team's collective military research experience, we created scale items to capture different types of recovery (e.g., musculoskeletal, immune). The MIIRS items were then shared with our active duty partners and another established psychometrician for feedback. Participants were asked to respond to each item on a scale from 0 (strongly disagree*)* to 4 (*strongly agree*). Some items were scored on a reverse scale (0 = strongly agree and 4 = strongly disagree). Example items include, “When I experience an injury such as an ankle sprain, I tend to recover quickly with adequate rest and/or rehabilitation,’’ and ‘‘When I get sick with the flu or a cold, it seems to take a very long time before I am able to get back to work.”

### Data collection

Participants were presented with a survey that included the MIIRS-17 and demographic questions as well as measures of pain, fatigue, occupational stress, depression, anxiety, sleep quality, resilience, and readiness. Participants were asked to rate their average pain with a single item on a scale from 0 (no pain) to 10 (as bad as it could be, nothing else matters) using the Defense and Veterans Pain Rating Scale (18). They were also asked to rate their fatigue in the past week on a numeric rating scale from 0 (no fatigue) to 10 (severe fatigue). Fatigue numeric rating scales have been shown to be valid and responsive measures in research (19). Occupational stress was assessed using a 4-item scale that was adapted from the Department of Defense Survey of Health-Related Behaviors [total possible range = 4–16 (20)]. Symptoms of depression and anxiety were measured with the Patient Health Questionnaire-8 [total possible range = 0–24 (21)] and the Generalized Anxiety Disorder-7 scale [total possible range = 0–21 (22)], respectively. Sleep quality and disturbances over the last one-month period were measured with the Pittsburgh Sleep Quality Index [PSQI; total possible range = 0–21 (23)].

The Connor-Davidson Resilience Scale, 10-item [CD-RISC-10 (24)] assesses overall resilience such as coping with, and recovering from, adversity. Items were rated on a 5-point scale (0–4), with higher scores reflecting greater resilience (total possible range = 0–40). The scale measures the ability to tolerate experiences such as change, personal problems, illness, pressure, and failure. Extensive evidence is available for the validity and reliability of the CD-RISC-10 (25).

The Military Readiness Scale, 15-item [MRS-15 (26)] assesses self-reported readiness across the three domains (general-psychological, technical, and physical readiness), which are combined to yield a total readiness score percentage (total possible range = 0%–100%). The MRS-15 is a novel measure developed by our team and was guided by our operational definition of readiness: the ability of a military service member to safely and effectively perform varied occupational duties to meet the diverse demands of assigned missions. Item responses ranged from 0 (strongly disagree) to 4 (strongly agree) and 0 = strongly agree and 4 = strongly disagree for reverse scored items. The MRS-15 is a valid measure with high reliability [Cronbach's *α* = 0.91 (26)].

### Data analysis

Participant characteristics were examined using means, standard deviations, and ranges. Factor structure of the recovery scale was explored with principal components analysis and Varimax rotation with Kaiser normalization. A Promax rotation was also conducted to explore methodological differences between rotations and to ensure that the most conservative rotation method was selected. The range, variance, and floor/ceiling effects of each candidate item were examined (acceptable range ≥ 3 and variance *SD* ≥ 0.6). Next, candidate items were loaded into an initial principal components factor model. The criteria for initial factor identification included an Eigen value > 1.0 and a substantial contribution (≥ 5.0%) to the total latent construct (i.e., recovery). The initial criteria for item retention were decisive loading on a single factor (> 0.5) with low cross loading (≤ 0.5) onto other factors. Within factors, each item was required to demonstrate a substantial corrected item-total correlation (≥ 0.5) and *α* ≥10% reduction of Cronbach *α*, if the item was removed. Each factor was considered internally reliable if Cronbach *α* ≥0.70. For every instance in which an item was removed, the principal components model was repeated until we derived a final, optimized solution. We observed negligible differences between Varimax and Promax rotations for the final item loadings and the rotation type did not change the subscale loadings. Subscales were named based on item commonality and theoretical precedence. The total recovery score and subscale scores were calculated as percentages (sum of [total or subscale] items divided by the maximum possible sum x 100%).

Convergent validity of the scale was established with bivariate associations between theoretically relevant correlates, total recovery score, and subscales scores. Model adjustments were made if a candidate covariate (e.g., age) related to the correlate and to recovery scores [both *p*s < 0.05 (27)]. Alpha was set at 0.05. To reduce the chance of a Type I error, Bonferroni adjustments were made for multiple bivariate correlations (*α* = 0.05/8 tests for each theoretically relevant external correlate = 0.006). For each bivariate comparison, we used nonlinear models (i.e., logarithmic, inverse, quadratic and/or sigmoidal) to explore improved data fit. Lastly, an age-adjusted multiple linear regression model was used to establish the unique and combined contributions of each predictor (e.g., pain) to total recovery. We considered a variance inflation factor (VIF) >5.0 as high, indicating a strong correlation between predictors, and this value was established as a cut-off for multicollinearity (28). In the analyses, we used available, complete observations for the variables in each model and we did not perform any imputations. All statistical models were conducted by the anchor author using SPSS (Version 29; IBM, Armonk, NY) and then independently reproduced, and verified, by one co-author and the first author.

## Results

Participant characteristics are outlined in Table 1. Most participants were male, White, enlisted (59.3% E4–E6) aircrew who were 20–29 years old (52.7%). Many had never deployed (33.5%) or been on a combat deployment (62.1%). The remainder of the sample had at least one deployment with some endorsing 4 or more deployments (24.2%) or combat deployments (3.8%).

**Table 1:** Participant characteristics.

Characteristic	*n* (%)	*M* ± *SD*	Range
Sex			
Male	175 (96.2)		
Female	7 (3.8)		
Race and ethnicity			
American Indian or Alaska Native^a^	0 (0.0)		
Asian	4 (2.2)		
Black or African American	5 (2.7)		
Hispanic or Latino	12 (6.6)		
Native Hawaiian or Pacific Islander	6 (3.3)		
Middle Eastern or North African^a^	0 (0.0)		
White	126 (69.2)		
Multiracial and multiethnic	29 (15.9)		
Age (years)		29.2 ± 6.9	19–48
Naval enlisted classification code or officer designator code			
Aircrew	134 (73.6)		
Pilot	38 (20.9)		
Other	10 (5.5)		
Military experience (years)	182	8.4 ± 6.2	<1–29
Military Illness and Injury Recovery			
Total	179	65.5 ± 14.9%	16.2–100.0%
Immune recovery	179	70.5 ± 18.3%	0.0–100.0%
Musculoskeletal injury recovery	180	59.1 ± 22.4%	0.0–100.0%
Physical recovery	180	65.8 ± 16.6%	6.3–100.0%
Rehabilitation response	180	64.4 ± 18.8%	0.0–100.0%
Pain	182	3.3 ± 2.0	0–10
Fatigue	182	4.7 ± 2.1	0–10
Occupational stress	182	8.9 ± 2.6	4–16
Depressive symptoms	181	6.2 ± 5.5	0–24
Anxiety symptoms	182	6.1 ± 5.9	0–21
Sleep disruption	174	7.9 ± 4.1	1–21
Resilience	180	32.6 ± 5.1	16–40
Military Readiness			
Total	181	75.0 ± 16.0%	23.3–100.0%
General–psychological	181	76.5 ± 17.8%	11.1–100.0%
Technical	181	74.5 ± 18.8%	8.3–100.0%
Physical	181	71.0 ± 22.9%	0.0–100.0%

*M*, mean; *SD*, standard deviation.

a Seven people identified as American Indian or Alaska Native, and two people identified as Middle Eastern or North African, but all nine also identified with at least one additional race/ethnicity.

All candidate recovery scale items demonstrated sufficient range (≥ 3 out of 4). We observed few floor effects (0.6%–8.3%) and modest ceiling effects (7.2%–29.4%). Removal of two items with complex factor loadings yielded a highly consistent, 17-item scale (Cronbach *α* = 0.90). A scree-plot revealed 4 factors (Eigen values > 1.0) thus, we derived a total recovery score (17 items) as well as four subscale scores, which we termed immune recovery (6 items, *α* = .87), musculoskeletal injury recovery, (MSKI; 4 items, *α* = .87), physical recovery (4 items, *α* = .72), and rehabilitation response (3 items, *α* = .67). There were minimal floor (0%–0.6%) and ceiling effects (0.0%–6.7%) at the total or subscale level. In the 17-item rotated factor matrix (Table 2), the four-factor solution accounted for 65.5% of variance in the total recovery construct. Low-moderate correlations between the subscales were observed (*r* range = .40–.54, all *p* < .001). The MIIRS-17 and scale scoring guide are included in the Appendix and a fillable form with automatic scoring is available (Supplementary Material).

**Table 2:** Rotated factor matrix.

Scale item (abbreviated description)		Component			
		1	2	3	4
1.	Sick with flu or cold, takes longer than most to recover	**0** **.** **70**	0.21	0.29	−0.06
2.	When I bruise, remains sore for a long time	**0**.**64**	0.42	0.02	0.12
3.	Flu shots always knock me down for several days	**0**.**72**	−0.01	−0.06	0.31
4.	Sick with flu or cold, always takes long time to get back to work	**0**.**78**	0.20	0.12	0.07
5.	When I bruise, seems to take forever to heal	**0**.**81**	0.24	0.06	0.14
6.	Other people seem to recover from illness faster than I do	**0**.**79**	0.15	0.28	−0.02
7.	Muscle or joint sprain becomes nagging, recurring injury	0.16	**0**.**89**	0.16	0.12
8.	Muscle or joint sprain, experience persistent pain afterwards	0.17	**0**.**83**	0.24	0.14
9.	No amount of rest or rehabilitation helps me regain performance	0.35	**0**.**56**	0.02	0.37
10.	Muscle or joint sprain, tend to reinjure frequently	0.27	**0**.**73**	0.23	0.10
11.	Injury such as ankle sprain, tend to recover quickly	0.10	0.38	**0**.**55**	0.15
12.	Exercise intensely, can exercise again the next day	0.08	0.18	**0**.**78**	0.03
13.	After injury, require less rest and medication than most	0.44	−0.02	**0**.**56**	0.18
14.	After medical procedures, tend to bounce back better than ever	0.12	0.13	**0**.**71**	0.28
15.	Respond to rehabilitation exercises well and recovery quickly	−0.03	0.23	0.47	**0**.**58**
16.	For most injuries, no amount of rehabilitation seems to be enough	0.29	0.39	0.04	**0**.**67**
17.	For most injuries, rest and rehabilitation bring me back to my old self	0.09	0.06	0.35	**0**.**76**

Bold values indicate factor loadings > 0.5.

Bivariate associations between MIIRS-17 subscales and theoretically relevant correlates are shown in Table 3. Age was related to pain, occupational stress, sleep disruption, resilience, and military readiness. Age was also associated with total recovery (*r* = −.34, *p* < .001) and all four subscales (immune recovery, *r* = −.25, *p* < .001; MSKI recovery, *r* = −.27, *p* < .001; physical recovery, *r* = −.36, *p* < .001; rehabilitation response, *r* = −.19, *p* = .01). In subsequent models, we adjusted for age accordingly. Total recovery was positively associated with resilience and military readiness and inversely associated with the remaining correlates (all *p*s < .001). We did not observe improvements in data fit with the application of nonlinear models (e.g., logarithmic, inverse).

**Table 3:** Relationships between recovery scores and theoretically related correlates.

Recovery Score	Total	Immune	MSKI	Physical	Rehab
Pain^a^	−.36***	−.22**	−.45***	−.16*	−.28***
Fatigue	−.27***	−.20*	−.32***	−.14	−.21**
Occupational stress^a^	−.28***	−.20*	−.30***	−.23**	−.16*
Depressive symptoms	−.34***	−.20*	−.34***	−.23**	−.35***
Anxiety symptoms	−.34***	−.21*	−.34***	−.23**	−.32***
Sleep disruption^a^	−.41***	−.32***	−.39***	−.25**	−.30***
Resilience^a^	.47***	.32***	.33***	.51***	.35***
Military readiness^a^	.47***	.29***	.39***	.43***	.41***

Bonferroni corrected *α* = .006.

a Age-adjusted.

*p < .05.

**p < .006.

***p < .001.

In the combined model, we found that the eight predictors and the covariate (age) explained 39.6% of variance in military recovery (*F*(9,170) = 13.4, *p* < .001). None of the predictors met criteria for multicollinearity (all VIFs < 5.0). In the overall model, sleep disruption (*b* = −.22, *p* < .05), resilience (b = .26, *p* = .001), and age (*b* = −.17, *p* < .05), independently contributed to recovery.

## Discussion

In this report, we describe the development and initial validation of the MIIRS, a 17-item self-report measure of recovery. We found the scale to be highly consistent and the principal component models demonstrated theoretical space for a stable, four-factor solution, which resulted in four subscales (immune recovery, MSKI recovery, physical recovery, and rehabilitation response). The high internal consistency across the 17 items indicated that the MIIRS-17 can measure self-perceptions of recovery. This is aligned with the results of other studies that self-perceptions of recovery can be reliably measured (9, 11). The correlations between subscales revealed that each subscale represented a related and unique facet of the same construct (recovery) with sensitivity to the individual contributions of each type of recovery (as represented by the four subscales). Upon further validation, the MIIRS-17 could be used to support recovery interventions and the development of health/MSKI risk trajectory models across the military career (29). Of note, since TBI is a concerning military issue, there is some evidence that the severity of a TBI can mediate self-perceptions (12). Thus, more extensive validation is needed to determine whether this self-report scale functions comparably in patients with different TBI classifications (i.e., mild, moderate, severe).

We observed evidence of convergent validity in the relationships between the MIIRS-17 and theoretically relevant correlates. Per our hypotheses, total recovery was inversely associated with pain, fatigue, mental health symptoms, and sleep disruption, but positively related to resilience and military readiness. The associations between total recovery and these correlates also indicated that recovery is a multivariate, but distinctly unique, construct.

In the multiple linear regression model, eight predictors (e.g., pain, fatigue) combined with age to explain almost 40% of the variance in recovery. Sleep disruption and age were independent, negative predictors of recovery. Sleep quality and quantity are integral not only to physiological and psychological recovery (30), but also to immune health (31). Age is also a well-established negative predictor of recovery. A reduced capacity to recover is linked to diminished biological resources (e.g., stem cells) and repair that is both delayed and inadequate (31). Interestingly, self-perceptions of aging have been reported to predict recovery independent of other anticipated moderators [i.e., age, sex (32)], further illustrating the complex relationship between psychological and physical health. We also recognize that age-related factors that were not measured in our study (e.g., chronic disease) could partially explain the association between age and recovery. In contrast, resilience was an independent, positive predictor of recovery in our study. This is aligned with evidence that the ability to recover is a form of biological resilience (31), which can be measured in processes such as immune function and muscle repair. Together, the independent predictors of recovery that we identified enhance the convergent validity of the MIIRS-17. This highlights the utility of the MIIRS-17 to potentially accelerate applied research and inform the development of new interventions (e.g., a new treatment for MSKI) and rehabilitation strategies.

### Limitations

Our results were derived from a research sample, not a clinical population and thus, this work is merely the first step to establish the MIIRS-17 as a clinical tool. While we retained all data from female participants in our analyses, the sample was mostly male so our results may not be generalizable to females. Further, this sample was comprised of only U.S. Navy aircrew and our findings may not apply to other military or non-military populations (e.g., veterans, clinical populations). To mitigate the risk of response bias with methods of self-report, we assigned participants non-traceable study identification numbers to facilitate honest survey responses. We did not explore the effects of item valence (e.g., positive vs. negative) in the MIIRS-17, and this should be tested in future studies. We also found the internal reliability of the rehabilitation response subscale to be slightly below our self-imposed criteria. We attribute this to the small number of items that loaded onto this subscale and to the overall preliminary nature of this study. Lastly, the predictive validity of the MIIRS-17 has not yet been established, but this is a current effort in our lab.

## Conclusions

This study preliminarily yields a valid and reliable measure to quickly and cost-effectively assess self-perceptions of recovery in military members. The MIIRS-17 is a promising tool to further understand individual recovery perceptions. Following more extensive validation, the MIIRS-17 could be used to quantitatively assess a patient's recovery propensity in a clinical setting. Thus, the MIIRS-17 may be a useful measure to support recovery interventions, to potentially predict recovery outcomes, and to assist in the development of risk models across years of service for military members and populations.

## Data Availability

The datasets generated and/or analyzed during the current study are not publicly available since public deposition would breach compliance with the protocol approved by the Naval Health Research Center Institutional Review Board. Data may be made available upon reasonable request and approval by the Naval Health Research Center Institutional Review Board/Privacy Office (usn.point-loma.navhlthrschcensan.list.admin@health.mil).

## Author disclaimer

AV, KH, JC, JH, and MT are military service members or employees of the U.S. Government. This work was prepared as part of their official duties. Title 17, U.S.C. §105 provides that copyright protection under this title is not available for any work of the U.S. Government. Title 17, U.S.C. §101 defines a U.S. Government work as work prepared by a military service member or employee of the U.S. Government as part of that person's official duties. Report No. 26-04 was supported by the Defense Health Agency under work unit no. 62362. The views expressed in this article are those of the authors and do not necessarily reflect the official policy or position of the Department of the Navy, Department of Defense, nor the U.S. Government. The study protocol was approved by the Naval Health Research Center Institutional Review Board in compliance with all applicable Federal regulations governing the protection of human subjects. Research data were derived from an approved Naval Health Research Center Institutional Review Board protocol, number NHRC.2023.0005.

## Appendix

Military Illness and Injury Recovery Scale-17 (MIIRS-17)

**Table:**

Indicate how much you agree, or disagree, with the statements below.		Strongly disagree	Disagree	Neither agree nor disagree	Agree	Strongly agree
1.	When I experience an injury such as an ankle sprain, I tend to recover quickly with adequate rest and/or rehabilitation.	0	1	2	3	4
2.	When I get sick with the flu or a cold, it usually takes me longer than most people to feel healthy again.	0	1	2	3	4
3.	Once I sustain a muscle or joint sprain, it usually becomes a nagging, recurring injury.	0	1	2	3	4
4.	I respond to rehabilitation exercises well and I recover quickly.	0	1	2	3	4
5.	Once I sustain a muscle or joint sprain, I usually experience nagging, persistent pain afterwards.	0	1	2	3	4
6.	When I bruise, it remains sore for a long time.	0	1	2	3	4
7.	When I exercise intensely, I am usually able to exercise hard again the following day without much rest.	0	1	2	3	4
8.	For most injuries that I’ve experienced, no amount of rehabilitation seems to be enough to get back to where I was before the injury.	0	1	2	3	4
9.	Flu shots, or other similar vaccinations, always knock me down for several days.	0	1	2	3	4
10.	After a significant physical injury, I tend to require less rest and medication than most people do.	0	1	2	3	4
11.	It seems that no amount of rest or rehabilitation helps me regain my performance at work after I am injured.	0	1	2	3	4
12.	For most injuries that I have experienced, rest and/or rehabilitation bring me back to my old self rather quickly.	0	1	2	3	4
13.	When I get sick with the flu or a cold, it seems to take a very long time before I am able to get back to work.	0	1	2	3	4
14.	Once I sustain a muscle or joint sprain, I tend to reinjure it more frequently than the average person does.	0	1	2	3	4
15.	After medical procedures, I tend to bounce back better than ever.	0	1	2	3	4
16.	When I bruise, it seems to take forever to heal.	0	1	2	3	4
17.	I’ve noticed that other people around me seem to recover from illness faster than I do.	0	1	2	3	4

MIIRS-17 Scoring Guide^a^

- Total Recovery percent score = [Sum of all 17 items/68] x 100%.
- Subscale percent scores = (sum score of numbered items) divided by (/) the total number of subscale items multiplied (x) by 100.
  “rev” = indicates a reverse-scored item (0 = 4; 1 = 3; 2 = 2; 3 = 1; 4 = 0).
  ○ Immune Recovery = ([2rev + 6rev + 9rev + 13rev + 16rev + 17rev])/24) x 100.
  ○ Musculoskeletal Injury Recovery = ([3rev + 5rev + 11rev + 14rev]/16) *100.
  ○ Physical Recovery = ([1 + 7 + 10 + 15]/16) *100.
  ○ Rehabilitation Response = ([4 + 8rev + 12]/12) *100.

^a^MIIRS-17 normative scores have not yet been established.
