# Supplementary material for: A novel assessment of self-perceptions of recovery from illness and injury in U.S. Navy service members
Source: Front Rehabil Sci. 2026 Sep 9;7:1846239. doi: 10.3389/fresc.2026.1846239 (PMC13599404; doi:10.3389/fresc.2026.1846239)
Supplement: Supplementary file 1 [file Supplementaryfile1.pdf]

Military Illness and Injury Recovery Scale-17 item (MIIRS-17)

**Indicate how much you agree, or disagree, with the statements below.**	Strongly disagree	Disagree	Neither agree nor disagree	Agree	Strongly agree
1. When I experience an injury such as an ankle sprain, I tend to recover quickly with adequate rest and/or rehabilitation.	○	○	○	○	○
2. When I get sick with the flu or a cold, it usually takes me longer than most people to feel healthy again.	○	○	○	○	○
3. Once I sustain a muscle or joint sprain, it usually becomes a nagging, recurring injury.	○	○	○	○	○
4. I respond to rehabilitation exercises well and I recover quickly.	○	○	○	○	○
5. Once I sustain a muscle or joint sprain, I usually experience nagging, persistent pain afterwards.	○	○	○	○	○
6. When I bruise, it remains sore for a long time.	○	○	○	○	○
7. When I exercise intensely, I am usually able to exercise hard again the following day without much rest.	○	○	○	○	○
8. For most injuries that I've experienced, no amount of rehabilitation seems to be enough to get back to where I was before the injury.	○	○	○	○	○
9. Flu shots, or other similar vaccinations, always knock me down for several days.	○	○	○	○	○
10. After a significant physical injury, I tend to require less rest and medication than most people do.	○	○	○	○	○
11. It seems that no amount of rest or rehabilitation helps me regain my performance at work after I am injured.	○	○	○	○	○
12. For most injuries that I have experienced, rest and/or rehabilitation bring me back to my old self rather quickly.	○	○	○	○	○
13. When I get sick with the flu or a cold, it seems to take a very long time before I am able to get back to work.	○	○	○	○	○
14. Once I sustain a muscle or joint sprain, I tend to reinjure it more frequently than the average person does.	○	○	○	○	○
15. After medical procedures, I tend to bounce back better than ever.	○	○	○	○	○
16. When I bruise, it seems to take forever to heal.	○	○	○	○	○
17. I've noticed that other people around me seem to recover from illness faster than I do.	○	○	○	○	○

FINISH

Total Military Recovery Score:

Immune Recovery:
Musculoskeletal Injury Recovery:
Physical Recovery:
Rehabilitation Response:

Hernández LM, Tovey NR, Venkatesh A, Heaton KJ, Campbell JS, Hru JM and Taylor MK (2026) A novel assessment of self-perceptions of recovery from illness and injury in U.S. Navy service members. Front. Rehabil. Sci. 7:1846239.
